# Immediate Effect of Whole-Body Vibration Exercise Performed in Vertical Versus Side-Alternating Displacement Modes on Physiological Parameters, Perception of Effort, Strength and Functionality in Adults with Obesity

**DOI:** 10.3390/diagnostics16020316

**Published:** 2026-01-19

**Authors:** Aline Reis-Silva, André Luiz Bandeira Dionizio Cardoso, Ana Carolina Coelho-Oliveira, Daniel Batouli-Santos, Gabriel Siriano Damasceno dos Santos, Jennyfer Silva Mazini, Ana Gabriellie Valério-Penha, Alessandra Andrade-Nascimento, Marcia Cristina Moura-Fernandes, Redha Taiar, Alessandro Sartorio, Danúbia da Cunha de Sá-Caputo, Mario Bernardo-Filho

**Affiliations:** 1 Programa de Pós-Graduação em Ciências Médicas, Universidade do Estado do Rio de Janeiro, Rio de Janeiro 20550-170, RJ, Brazil; 2 Laboratório de Vibrações Mecânicas e Práticas Integrativas, Departamento de Biofísica e Biometria, Instituto de Biologia Roberto Alcantara Gomes, Policlínica Universitária Piquet Carneiro, Universidade do Estado do Rio de Janeiro, Rio de Janeiro 20950-003, RJ, Brazildanielbatouli@gmail.com (D.B.-S.); bernardofilhom@gmail.com (M.B.-F.); 3 Programa de Pós-Graduação em Fisiopatologia Clínica e Experimental, Universidade do Estado do Rio de Janeiro, Rio de Janeiro 20551-030, RJ, Brazil; 4 Department of Sport Sciences, MATériaux et Ingénierie Mécanique (MATIM), Université de Reims, 51687 Reims, France; 5 Experimental Laboratory for Auxo-Endocrinological Research, Istituto Auxologico Italiano, Istituto diRicovero e Cura a Carattere Scientifico (IRCCS), 28824 Piancavallo-Verbania, Italy; sartorio@auxologico.it

**Keywords:** obesity, vibration, vital signs, physical functional performance

## Abstract

**Background**: Obesity, defined as an abnormal accumulation of body fat, is becoming a global epidemic. Individuals with obesity may present with increased abdominal fat, which is associated with hypertension, altered respiratory mechanics, higher resting heart rate, and may contribute to an increased cardiovascular risk. Physiological parameters, such as heart rate, blood pressure, respiratory rate, and oxygen saturation, can change hours before the occurrence of a clinically relevant adverse event. Thus, physiological parameters can be considered good predictors of clinical deterioration. Obesity is also associated with physical dysfunctions that can impair physical performance. The non-pharmacological therapeutic strategy for the treatment of obesity involves lifestyle modifications, including a healthy diet and regular physical exercise. Whole-body vibration (WBV) exercise, a type of physical activity, has demonstrated benefits in several specific populations, including obese individuals. Objectives: The objective of this study was to evaluate the immediate effects of a single whole-body vibration (WBV) exercise session, consisting of 15 sets, using a vibration platform (VP) with alternating vertical or lateral displacement, on physiological parameters, perceived exertion, strength, and functionality in obese adults. **Methods**: Seventy-two obese adult participants were randomly divided into three groups (vertical group, alternating lateral group, and placebo group). Physiological parameters were assessed before, during, and after the intervention, in addition to perceived exertion, functionality, and muscle strength. **Results**: When comparing the results before and after the intervention, the heart rate–pressure product increased significantly in the alternating lateral group (*p* = 0.005), and heart rate increased significantly (*p* = 0.0001) and then decreased significantly (*p* = 0.030) only in the alternating lateral group. Post hoc analysis revealed a significant increase in perceived exertion in the lateral alternation group, from the period before the intervention to the 10th set (*p* = 0.006) and from the period before to the period after the intervention (*p* = 0.011). In the vertical group, a significant increase was observed from the period before the intervention to the 10th set (*p* = 0.020). **Conclusions**: In conclusion, considering all the findings of this study, whole-body vibration (WBV) exercise promoted some immediate changes in physiological parameters and perception of effort in obese adults. WBV exercise with the alternating vibration platform induced significant fluctuations in heart rate and increased the heart rate–blood pressure product, although with values within the normal range. Perception of effort increased in all groups. Considering the absence of discrepant changes in physiological parameters, impact on the cardiovascular system, and fatigue, the WBV exercise intervention in side-alternating or vertical vibration vibratory platforms can be considered a viable non-conventional exercise option for the obese population.

## 1. Introduction

Obesity is a health condition characterized by excessive accumulation of body fat, often resulting from an interplay of genetic, behavioral, and environmental factors. Its global prevalence has been steadily increasing, accompanied by a rise in the incidence of associated comorbidities [1], such as hypertension. [2]. Notably, the prevalence of hypertension among obese individuals with a BMI ≥ 30 kg/m^2^ is 42.5%, compared to 15.3% in non-obese individuals. This increase in the prevalence of hypertension among individuals with obesity may be attributed to the accumulation of fat in the abdominal region, mainly related to visceral fat, which is a strong determinant of elevated BP levels [3].

Abdominal and thoracic fat deposition, in addition to affecting BP levels, may also lead to a reduction in respiratory capacity [4]. This fat accumulation compresses and impairs diaphragmatic and thoracic mobility, leading to changes in lung compliance and respiratory resistance, which ultimately reduce lung volume and increase respiratory rate (RR) and dyspnea [5]. Dyspnea is a common symptom in individuals with obesity and may be associated with body mass gain, even in the absence of respiratory diseases [6]. Furthermore, Garg et al. (2016) [7] suggest that increased abdominal circumference is a negative regulator of arterial oxygen saturation (SpO_2_) in both diabetic and non-diabetic obese patients. Therefore, obesity could be considered a contributing factor to hypoxia [7].

Obesity is also associated with various cardiovascular health problems such as coronary artery disease and heart failure [8]. Heart rate (HR) is commonly used as an indicator of cardiovascular function, reflecting the heart’s ability to respond to metabolic demands [9]. Studies have shown that individuals with obesity may present a higher resting HR compared to non-obese individuals [10,11], possibly due to increased metabolic demand and autonomic imbalance with enhanced sympathetic activity [12]. In this context, individuals with obesity tend to report a higher perceived effort during physical exercise (PE) compared to lean individuals and oxidize less body fat during submaximal exercise [13]. Therefore, monitoring perceived exertion in these individuals and developing PE strategies to increase fat oxidation and reduce perceptions of discomfort during PE may improve exercise tolerance and adherence and facilitate fat mass reduction [14].

In addition to its influence on the cardiovascular system, obesity is also associated with sarcopenic obesity, characterized by low muscle mass and high body fat, which impairs musculoskeletal tissue in both youth and older adulthood [15]. The implications of reduced muscle strength relative to body mass in the lower limbs may contribute to impaired physical performance, including slower walking speed, increased risk of falls, and a higher risk of joint diseases [16]. Aging and obesity progression also contribute to muscle quality decline due to intramuscular fat accumulation [17].

According to studies, changes in physiological parameters such as HR, BP, RR, and SpO_2_ occur several hours before an important adverse event takes place [18]. Therefore, physiological parameters can predict deterioration hours before clinical adverse events [19]. Moreover, they represent simple and low-cost information collected at the bedside, in outpatient settings, or in research environments, identifying health deterioration, management responses to clinical situations, and monitoring interventions involving PE [20].

Regular PE improves cardiovascular and metabolic function, muscle mass, strength, and physical performance in obesity [21]. PE benefits involve multifactorial mechanisms, including energy balance regulation [22]. However, barriers such as poor fitness, joint pain, and social embarrassment reduce adherence to traditional PE programs in obese individuals [23]. Therefore, it would be useful to develop and study PE alternatives that can positively and safely encourage adherence in this population to PE [24].

Whole-body vibration (WBV) exercise is generated in an individual exposed to mechanical vibration produced in a vibrating platform (VP) during systemic vibratory therapy (SVT) [25]. WBV exercise has been reported as a modality of PE for various populations, including individuals with obesity [26]. In general, individuals appear to have good adherence to WBV exercise because the exercises can be easily performed with rest periods between sets and potentially lower perceived exertion [27].

The VP promotes SVT to the entire body through two different transmission systems of mechanical vibrations: (i) vertical VP, where the base of the VP performs uniform vertical displacements, moving up and down, and (ii) side-alternating VP, where the base of the VP performs alternating lateral displacements, like a seesaw [28].

The posture adopted (standing, sitting), the type of exercise (dynamic or static), work and rest time, and the number of sets performed, as well as the intensity of the vibratory stimulus defined by the formula a Peak = 2 × π^2^ × f^2^ × PPD (a Peak = peak acceleration in m/s^2^; f = frequency in hertz; PPD = peak-to-peak displacement in millimeters), may vary considerably and influence the intended intervention effect [29].

In SVT, the vibratory stimulus is transmitted to muscles and tendons during WBV exercise. Consequently, a cyclic transition between eccentric and concentric muscle contractions occurs, leading to a neuromuscular response of chronic adaptations to the intervention effect through muscle spindles and rapidly adapting mechanoreceptors, potentially resulting in improved neuromuscular performance [30].

Immediate WBV exercise protocols may contribute to improvements in flexibility, physical performance, and increased oxygen uptake [31]. Although WBV exercise appears to be a promising modality for enhancing physical performance, there is a gap in the literature regarding conclusive evidence on immediate-effect protocols of WBV exercise using two different types of vibration platforms and investigating physiological parameters, perceived exertion, and functionality in obese participants. Therefore, the aim of this study was to assess the immediate effects of a single WBV exercise session using vertical and side-alternating vibrating platforms on physiological parameters, perceived exertion, strength, and functionality in adults with obesity. The hypothesis is that a single session of whole-body vibration (WBV) exercise, performed on a vertical or side-alternating vibration platform, may promote changes in physiological parameters, functionality, and perceived exertion in obese adults.

## 2. Methods

### 2.1. Study Design/Participants

The current randomized, interventional study, with participant and outcome assessor blinding, was conducted at the Laboratório de Vibrações mecânicas e Práticas Integrativas (LAVIMPI), Universidade do Estado do Rio de Janeiro, from 2022 to 2024. The protocol was approved by the ethics committee under registration number CAAE 30649620.1.0000.5259 and approved on 6 May 2020 [32]. Participants were recruited from the endocrinology department of the Policlinica Universitária Piquet Carneiro, Universidade do Estado do Rio de Janeiro, and from the general community between October 2022 and October 2023. Potential participants were identified through consultation of service records and dissemination in the community through posters and social networks.

Initial contact was made by telephone by a previously trained researcher, who provided detailed information about the objectives, procedures, and duration of the study. During this contact, an initial screening was conducted to verify the main eligibility criteria. All participants signed the informed consent form, and the procedures were performed at LAVIMPI in the morning.

### 2.2. Randomization and Masking

In this study, sample randomization was performed using the online randomization tool available at https://www.random.org/lists/ [33]. This website generated a numbered random sequence list containing three groups: (1) vertical group; (2) side-alternating group; and (3) sham group (SG). A researcher not involved in the collection prepared opaque, sealed and numbered envelopes, each containing the group allocation and trial order, randomized accordingly.

On the day of randomization, each participant drew a numbered, sealed envelope and handed it to the researcher responsible for administering the intervention. Participants had no access to information regarding their group allocation. All results were recorded anonymously using participant identification numbers and were transferred to the blinded statistical analysis team prior to final processing. Both the participants and the statistician were blinded to allocation. All assessments were performed in a controlled laboratory setting, maintaining ambient conditions (22–24 °C).

### 2.3. Eligibility Criteria

Eligible patients were men and women aged 18 to 59 years with a diagnosis of grade 1 or grade 2 obesity (BMI between 30 and 39.9 kg/m^2^), as defined by the World Health Organization (WHO) criteria [34], and who provided written informed consent. The age range and BMI were defined to ensure greater homogeneity within the group.

The exclusion criteria were considered to be individuals with BP levels above BP ≥ 180/110 mmHg, cardiovascular diseases manifested in the last 6 months, pregnant women, abuse or use of psychiatric medications, cardiac pacemaker wearers, any disease that prevents the individual from maintaining the orthostatic position in VP and individuals who had any contraindication to the protocols of the intervention.

Study procedures, including assessments and interventions, were as follows: All participants were instructed to refrain from engaging in any strenuous physical activity for 48 h prior to the intervention. None reported any cardiorespiratory or musculoskeletal problems that could interfere with the protocol.

### 2.4. Procedures

#### 2.4.1. Outcome Measures

Anamnesis: During the anamnesis, data were collected regarding sex, age, history of chronic diseases (DM2, systemic arterial hypertension) and current use of medications, smoking habits, alcohol consumption, and whether the patient performs regular PE. These data were used to promote a greater understanding of the results obtained and to characterize the sample.

#### 2.4.2. Anthropometric Measurements

Anthropometric measurements were performed by experienced and trained researchers. Participants were instructed to stand in the standing position, and with the body areas to be evaluated undressed.

1. Height was measured using a stadiometer attached to an electronic scale (MIC 200 PPA, Micheletti, São Paulo, Brazil) [35];
2. Body mass was measured using an electronic scale of the bioelectrical impedance device (In Body 370, BIOSPACE, Seoul, Republic of Korea) [36];
3. The body mass index (BMI) was calculated by dividing the body mass (kg) by the height squared (m^2^) [37]. BMI was classified according to the WHO criteria: obesity class I, BMI between 30 and 34.9 kg/m^2^; obesity class II, BMI between 35 and 39.9 kg/m^2^ [38].
4. Waist circumference (WC) assessment was measured with non-stretchable, flexible tape at the midpoint between the last rib and the iliac crest [37]. WC > than 102 cm in men and greater than 88 cm in women indicates abdominal obesity [39]. All measurements were recorded by experienced staff to minimize variability, with each measurement taken three times and the average used for analysis [39].
5. Hip circumference (HC) was measured using a non-flexible tape measure (tape measure: 0.1 cm) at the level of the widest part of the gluteal region [40]. Values below 95 cm for men and 108 cm for women are recommended [41].
6. Calf circumference was measured on the right leg at the point of maximum circumference using a non-flexible tape (precision: 0.1 cm). Cutoff points of 32 cm for women and 34 cm for men are associated with greater predictive ability for reduced muscle mass in adults [42].
7. Neck circumference (NC) was measured with the participants standing, head in the Frankfurt horizontal plane, using a tape placed below the laryngeal prominence, perpendicular to the longest axis of the neck. Mean values of 39.5 cm (standard deviation [SD] ± 3.6) for men and 34.0 cm (SD ± 2.9) for women are reported; values above these thresholds are associated with increased cardiovascular risk [43].
8. Waist-to-hip ratio (WHR) was calculated by dividing the WC (cm) by the HC (cm). Cutoff points were 0.85 for women and 0.90 for men [44].
9. Waist-to-height ratio (WHtR) was calculated by dividing the WC (cm) by the height (cm); WHtR correlates well with body fat and predicts hypertension, a key complication of obesity. A cutoff point of 0.50 was used for both sexes to indicate increased metabolic risk [45].
10. Body composition was assessed using a Tetrapolar Electrical Bioimpedance Analyzer (In Body 370, BIOSPACE, Republic of Korea), following the manufacturer’s recommendations to ensure standardization [46]. Participants were instructed to fast for 4 h; refrain from PE for 12 h; avoid coffee or alcohol for 48 h prior to assessment; empty bladder; wear swimwear or light clothing; stand for 5 min before collection; and remove bracelets, watches, shoes, and socks [46]. Parameters analyzed included body fat mass (kg), fat-free mass (kg), metabolic basal task, and trunk fat percentage (%).

#### 2.4.3. Blood Pressure

Systolic blood pressure (SBP) and diastolic blood pressure (DBP) were measured using a properly calibrated digital sphygmomanometer (Omron HEM-RML31, Omron Healthcare, Kyoto, Japan).

The measurement was performed after a 5 min rest period. Participants were instructed to refrain from PE for at least 90 min and to avoid alcoholic beverages, coffee, food, or smoking within 30 min prior to assessment. At the time of measurement, participants were asked to empty the bladder and position their left arm at heart level, with the palm facing upwards, ensuring that clothing did not constrict the arm [47].

The cuff was selected according to arm circumference and positioned without gaps, 2–3 cm above the cubital fossa, with the middle of the inflatable bag centered over the brachial artery [48].

Measurements were performed three times with a 1 min interval. The average of three BP measurements was used for statistical analysis [48]. The cutoff points considered were (i) normal (systolic pressure < 120 mmHg and diastolic pressure < 80 mmHg), (ii) high (systolic pressure from 120 to 129 mmHg and diastolic pressure < 80 mmHg), (iii) stage 1 hypertension (systolic pressure from 130 to 139 mmHg or diastolic pressure from 80 to 89 mmHg), and (iv) stage 2 hypertension (systolic pressure ≥ 140 mmHg or diastolic pressure ≥ 90 mmHg) and/or use of antihypertensive medications [48].

#### 2.4.4. Heart Rate

HR was assessed using a calibrated digital sphygmomanometer (Omron HEM-RML31, Omron Healthcare, Kyoto, Japan) and during the intervention, HR was measured after the 5th and 10th sets using a portable digital pulse oximeter (G. Tech, OLED Graph (MD300C23), Beijing Choice Electronic Technology Co., Ltd., Beijing, China), and values were recorded in beats per minute (bpm).

Considering the normal reference range for HR, values between 60 and 100 bpm were regarded as within the physiological limits. HR was monitored at the following time points during the intervention: (i) before the intervention; (ii) after the 5th set; (iii) after the 10th set; and (iv) after the intervention [49].

#### 2.4.5. Mean Arterial Pressure, Rate–Pressure Product, and Pulse Pressure

Mean arterial pressure (MAP) reflects cardiac workload and organ perfusion. It is calculated as (SBP + 2 × DBP)/3. MAP represents the average arterial pressure throughout a complete cardiac cycle, encompassing both systole and diastole. Values well above the normal range may indicate cardiovascular stress and potential organ damage, whereas values below 60 mm Hg may indicate insufficient blood supply to the organs, with a risk of organ failure. The normal range varies between 70 and 110 mmHg [50].

The rate–pressure product (RPP) is considered an index marker of myocardial oxygen demand (MVO_2_). MVO_2_ may be a good predictor of cardiovascular disease. RPP is calculated as the product of SBP and HR [51].

Pulse pressure (PP) is a risk factor for the development of heart disease; an increase of just 10 mmHg in pulse pressure increases cardiovascular risk by up to 20%. The coefficient of PP variation can reflect the magnitude of blood pressure fluctuation within a cardiac cycle and was calculated using the formula PP = SBP − DBP. A pulse pressure less than 25% of systolic pressure is considered inappropriately low or narrow, while a pulse pressure greater than 100 is considered high or wide [52].

#### 2.4.6. Respiratory Rate

The RR assessment was conducted in a quiet room by an experienced health researcher with the patient comfortably seated in a chair. RR measurements were made by counting breaths, with the evaluator carefully monitoring chest movements for one minute using a digital stopwatch, without the volunteers being aware of the process, so that no changes in the respiratory pattern occurred.

The normal reference range for adults is typically 12–20 breaths per minute (bpm). Slow breathing was defined as RR < 12 bpm, and tachypnea as RR > 20 bpm [53]. RR was measured three times before starting the intervention and three times after completing the intervention. The average of these values was recorded and used in the analyses [53].

#### 2.4.7. Arterial Oxygen Saturation

SpO_2_ was measured using a portable pulse oximeter (G. Tech, OLED Graph (MD300C23), Beijing Choice Electronic Technology Co., Ltd.) placed on the second finger of the right hand, with nails free of polish. Values greater than 95% were considered normal [54]. Measurements were taken at four time points: before the intervention, after the 5th set, after the 10th set, and after the intervention. At the beginning and end of the intervention, participants rested in a seated position for 10 min before three measurements were recorded at 1 min intervals; the mean value was used for analysis.

#### 2.4.8. Perceived Exertion (BORG Scale)

The Borg Rating of Perceived Exertion (RPE) scale (0–10) was used to evaluate the participants’ subjective effort during the intervention. Participants were shown a printed scale and asked, “How tired are you?”, with responses ranging from 0 (no fatigue) to 10 (maximum fatigue) [55]. The scale was applied at four time points: before the intervention, after the 5th set, after the 10th set, and after the intervention.

#### 2.4.9. Handgrip Strength/Dynamometry

Handgrip strength (HGS) was assessed using the American Society of Hand Therapists (ASHT) protocol. Participants sat comfortably with their feet shoulder-width apart, shoulders slightly adducted, elbows at 90°, forearms in a neutral position, and wrists extended between 0° and 30° [56]. Three maximal grip attempts were performed per limb (right and left), each lasting three seconds, with a 1 min rest between attempts to prevent fatigue [57]. All measurements were recorded in kilograms, and the mean value of three repeated trials was used for subsequent analysis.”,Cutoff values vary by sex, age, body mass, and hand dominance [58,59].

#### 2.4.10. Functionality Assessment

To assess functionality and physical performance, participants underwent the Short Physical Performance Battery test (SPPB), conducted by an experienced researcher. The SPPB is a tool used to evaluate functional capacity in adults, comprising three test categories: static balance, gait speed, and muscle strength. It includes three standing balance tests (feet side by side, semi-tandem, and tandem positions), with the maximum score awarded if the participant maintains balance for 10 s in each test; a timed 3 m walk at normal pace, recording the best of two attempts; and a timed sit-to-stand test measuring the time to complete five repetitions of sitting and standing from a standardized chair, with arms crossed in front of the body. Performance in each category is scored from 0 to 4 points, with a total score ranging from 0 to 12. The test battery takes approximately 10 to 15 min and was performed before and after the intervention [60]. The total SPPB score classifies functional capacity as follows: 0–3 points, indicating incapacity or low functional capacity; 4–6 points, indicating low functional capacity; 7–9 points, indicating moderate functional capacity; and 10–12 points, indicating good functional capacity. The minimum clinically significant change for the SPPB is 0.5 points, with 1 point representing a substantial change [61].

For the analyses, the following were considered: (i) balance test: score; (ii) walking test: gait speed, displacement time, and score; (iii) sit-to-stand test: execution time and score; (iv) the total score of the three tests.

#### 2.4.11. Intervention Procedures

All procedures were performed in a single day. Participants received prior instructions regarding preparation for the collection day (e.g., clothing, fasting, exercise). Upon arrival at the laboratory, they signed the informed consent form. After signing, participants completed an anamnesis questionnaire covering health status (medications, diabetes, hypertension) and lifestyle habits (smoking, alcohol consumption, physical activity).

Participants were taken to a room with a controlled temperature for height and bioelectrical impedance analysis (BIA) measurements, followed by the collection of anthropometric data (waist circumference, neck circumference, hip circumference, and calf circumference) in the same room for the interventions.

Before the intervention, physiological parameters (BP, HR, RR, SpO_2_) and perceived exertion (Borg scale) were measured. Functional capacity (SPPB test) and muscle strength (manual dynamometry) were also assessed. During the intervention, HR, SpO_2_, and perceived exertion were monitored. After the intervention, assessments of BP, HR, RR, SpO_2_, Borg scale, SPPB test, and dynamometry were repeated.

##### Intervention

**Figure 1:**
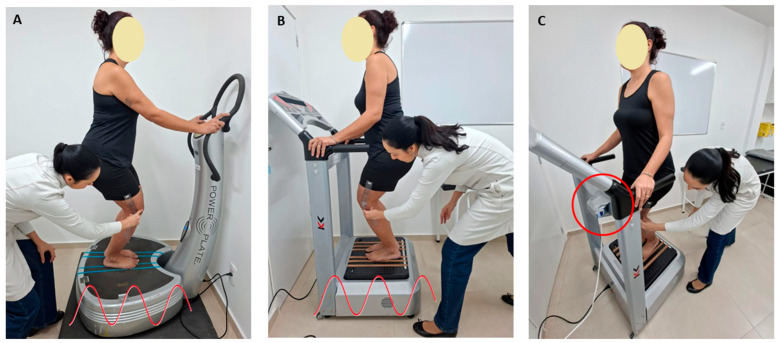
(**A**). Participant in the study being positioned by the researcher on the basis of the vertical VP in a static semi-squat posture, with the aid of a goniometer adjusted to 50° of knee flexion;The red line represents the sinusoidal wave produced by the vibrating platform during operation. (**B**). Participant in the study being positioned by the researcher on the basis of the side-alternating VP in a static semi-squat posture, with the aid of a goniometer adjusted to 50° of knee flexion; The red line represents the sinusoidal wave produced by the vibrating platform during operation. (**C**). Participant in sham group in the study being positioned by the researcher on the base of the side-alternating VP in a static semi-squat posture, with the aid of a goniometer adjusted to 50°, with VP turned off and and the red circle highlighting a device that emits a sound similar to that of a VP in operation.

Two different models of VP were used in the study: the vertical VP model (Power Plate Pro 5, North America, Inc., Irvine, CA, USA) [62] and the side-alternating VP (Kikos P204IX, Kikos Fitness, São Paulo, Brazil) [63]. The vertical VP was used by the vertical group, while the side-alternating VP was used by the side-alternating and sham groups, as shown in Figure 1A–C.

#### 2.4.12. Side-Alternating Group

Participants randomized to the side-alternating group were instructed to perform a static semi-squat with knees flexed to 50° (measured using a goniometer) at the base of the VP, remaining barefoot, standing, with the head in a neutral position, arms relaxed and lightly resting on the VP handles (Figure 1).

**Figure 2:**
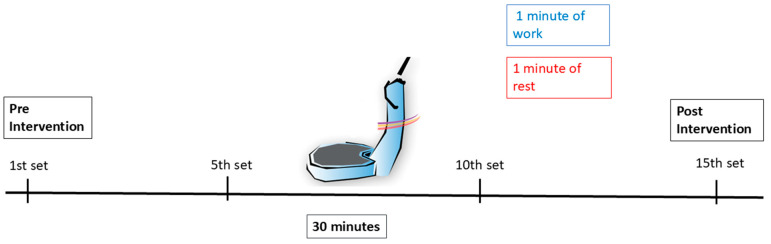
The timeline shows how the study protocol was performed from before intervention, through the 1 min work and 1 min rest periods, to the 5th set; from the 5th set to the 10th set; and from the 10th set to the end of the intervention, totaling 15th sets and 30 min of protocol.

Regarding biomechanical parameters, the VP frequency was set to 30 Hz on the display, and the second toe was positioned in line with the peak-to-peak displacement of 2.5 mm, corresponding to a distance of 2 cm between the feet (measured with a non-flexible tape measure), using the base of the hallux as an anatomical reference. Participants performed 15 sets of 1 min static squats (work time), alternating with 1 min of rest in a standing position between sets, as shown in Figure 2. The total session duration was 30 min.

#### 2.4.13. Vertical Group

Participants randomized to the vertical group were instructed to perform a static semi-squat with knees flexed at 50° (measured using a goniometer) at the base of the VP. They remained barefoot with a 2 cm distance between the feet (measured using a non-flexible tape measure), using the base of the hallux as an anatomical reference. They were in the standing position, with the head in a neutral position and arms relaxed, lightly resting on the VP handles—the same positioning used in the side-alternating group.

Regarding biomechanical parameters, the VP frequency was set to 30 Hz, and the peak-to-peak displacement was set to “low” on the display. Participants performed 15 sets of 1 min static squats, with a 1 min rest between each set. The total session duration was 30 min.

#### 2.4.14. Sham Group

Participants randomized to the sham group performed the same static semi-squat position with knees flexed at 50° (measured by a goniometer) at the base of the side-alternating VP. They were barefoot, with the second toe aligned with the peak-to-peak displacement of 2.5 mm, corresponding to a 2 cm distance between the feet (measured using a non-flexible tape measure), with the base of the hallux as an anatomical reference. They were in the standing position with the head neutral and arms relaxed, lightly supported on the VP handles. However, the VP remained stationary, despite the display being on. Instead, a device behind the VP emitted a sound mimicking VP operation, providing participants with a false sensation of WBV exercise. Participants completed 15 sequences of 1 min static semi-squats, with 1 min rest between sets. The total session time was 30 min.

### 2.5. Statistical Analysis

The sample size was calculated using G*Power^®^ 3.1.9.2 software (Franz Faul, Universität Kiel, Kiel, Germany) [64]. A power analysis was conducted using the repeated measures ANOVA within-between interaction test, based on Sá-Caputo et al. (2019) [65], using diastolic BP, with 85% power, 5% error probability, and an effect size of 0.15. The required total sample was 69 individuals (23 per group) [65].

Data processing and analyses were performed in R software version 4.4.2. Missing data were handled through multiple imputations using linear regression. Assumptions for multivariate analyses, including multivariate normality, homogeneity of variances and covariances, sphericity, and absence of multivariate outliers, were checked before hypothesis testing [66].

Baseline comparisons among groups were conducted using multivariate analysis of variance (MANOVA) for continuous variables and Fisher’s exact test for categorical variables. Handgrip strength was analyzed using a factorial repeated-measures ANOVA with group and time (pre- and post-test) as factors. Bonferroni-adjusted post hoc tests were applied when significant effects were found.

Vital signs (BP, HR, RR, SpO_2_) and perceived exertion were analyzed with mixed repeated measures ANOVA: moment (pre, moment 2, moment 3, post) as the within-subject factor and group (vertical, side-alternating, sham group) as the between-subject factor. The Greenhouse–Geisser correction was applied when sphericity was violated; Tukey post hoc tests were used to explore significant effects or interactions. SPPB scores were analyzed similarly using repeated-measures ANOVA to assess changes in functional performance over time and between groups.

Data are presented as mean ± standard deviation (continuous data), or as absolute and relative frequencies (categorical data). Statistical significance was set at a *p*-value of ≤0.05.

## 3. Results

### 3.1. Flowchart of the Study

**Figure 3:**
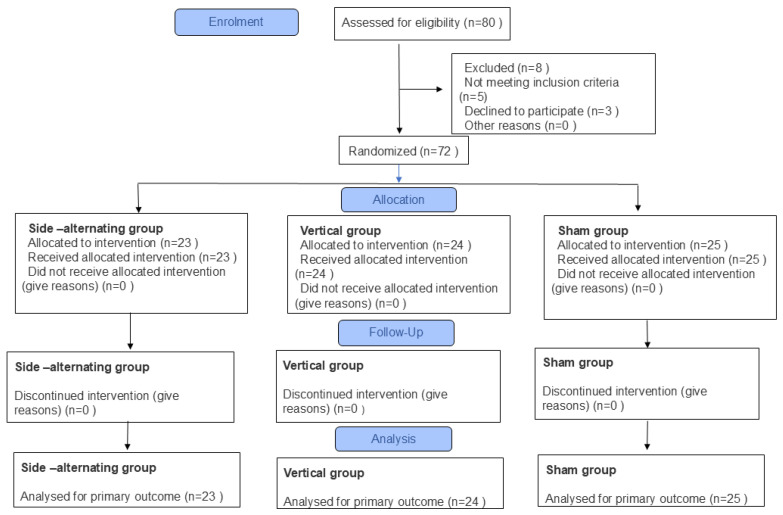
Flow diagram illustrating the number of participants in each group.

Eighty participants were recruited. Eight individuals did not meet the inclusion criteria: one volunteer was hypertensive with uncontrolled high BP; two volunteers had cardiovascular events in the past six months; two had a BMI above 39.9 kg/m^2^; and three declined to participate. Thus, seventy-two participants were randomized into three groups. As a result of randomization, 23 participants were assigned to the side-alternating group, 24 to the vertical group, and 25 to the sham group. There was no loss to follow-up during the study, possibly because the entire study was conducted in a single day. The study flowchart is shown in Figure 3.

### 3.2. Baseline Characteristics of Study Participants

Table 1 describes the characteristics of the 72 participants, including 20 males and 52 females, with an average age of 43.5 years. The mean age was 47.33 ± 8.23 years in the vertical group, 41.09 ± 9.37 years in the side-alternating group, and 42.08 ± 8.95 years in the sham group. All participants were individuals with obesity, with an average BMI of 34.72 kg/m^2^. The mean BMI was 34.79 ± 2.87 kg/m^2^ in the vertical group, 34.34 ± 2.94 kg/m^2^ in the side-alternating group, and 35.04 ± 2.64 kg/m^2^ in the sham group. Regarding BMI classification, 50% of participants were classified as obese grade I and 50% as obese grade II. Anthropometric variables, bioimpedance parameters, and lifestyle characteristics are also presented. Regarding chronic diseases associated with obesity, 27.7% of participants reported a medical diagnosis of hypertension, and 8.33% reported diabetes mellitus. There were no significant differences in demographic, anthropometric, or comorbidity profiles among the three groups.

### 3.3. Physiological Parameters

Table 2 presents the results for physiological parameters before and after the intervention in the three groups (vertical, side-alternating, and sham groups).

The analysis of BP showed no statistically significant differences in both intra- and intergroup comparisons for SBP (mmHg): vertical group (125.65 ± 15.72/126.93 ± 15.72), *p* = 0.278; side-alternating group (120.98 ± 13.79/120.82 ± 13.25), *p* = 0.919; sham group (120.98 ± 13.79/120.82 ± 13.25), *p* = 0.109; intergroup analysis, *p* = 0.321.

Similarly, DBP (mmHg) showed no significant intra- or intergroup differences: vertical group (82.69 ± 7.69/83.37 ± 7.15), *p* = 0.405; side-alternating group (80.50 ± 9.23/80.31 ± 8.42), *p* = 0.832; sham group (84.52 ± 11.60/83.42 ± 11.34), *p* = 0.401; intergroup, *p* = 0.419.

Mean arterial pressure (MAP, mmHg) also did not show significant intra- or intergroup changes: vertical group (183.08 ± 19.18/185.01 ± 18.61), *p* = 0.310; side-alternating group (177.08 ± 19.98/176.24 ± 18.11), *p* = 0.707; sham group (187.96 ± 23.20/183.24 ± 24.19), *p* = 0.239; intergroup, *p* = 0.311.

Regarding pulse pressure (PP), no significant differences were observed after the intervention in either intra- or intergroup analyses: vertical group (44.12 ± 14.95/45.91 ± 16.90), *p* = 0.411; side-alternating group (41.43 ± 11.84/42.47 ± 11.24), *p* = 0.558; sham group (45.76 ± 12.76/44.04 ± 12.65), *p* = 0.338; intergroup, *p* = 0.696.

However, the rate–pressure product (RPP) showed a significant increase only in the intragroup analysis of the side-alternating group after the intervention (8679.47 ± 1692.74/9395.86 ± 2273.57), *p* = 0.005. No significant changes were observed in the vertical group (9339.79 ± 1622.98/9443.45 ± 2070.50), *p* = 0.739; sham group (9589.16 ± 1819.02/9674.44 ± 1717.38), *p* = 0.751; or intergroup, *p* = 0.877.

Finally, RR showed no statistically significant changes: vertical group (17.68 ± 3.00/18.34 ± 2.89), *p* = 0.154; side-alternating group (15.53 ± 2.79/16.50 ± 3.34), *p* = 0.050; sham group (15.86 ± 4.51/16.21 ± 4.26), *p* = 0.352; intergroup, *p* = 0.086.

### 3.4. Vital Signs Measured During the Interventions

Table 3 presents the results regarding the physiological parameters (SpO_2_, HR, and Borg scale) assessed at four periods: (i) before the intervention [pre]; (ii) after the 5th set [moment 2]; (iii) after the 10th set [moment 3]; and (iv) after the intervention [post].

The analysis of SpO_2_ showed no statistically significant differences between groups (*p* = 0.229), across time points (*p* = 0.149; Greenhouse–Geisser-corrected), or for the group × time interaction (*p* = 0.649), indicating that SpO_2_ levels remained stable throughout the intervention period regardless of group allocation.

For the Borg scale of perceived exertion, a significant main effect of time (pre- to post- intervention) was observed (*p* < 0.001), with no significant differences between groups (*p* = 0.804) or group × time interaction (*p* = 0.824). Post hoc analysis revealed a significant increase in perceived exertion in the side-alternating group from pre to moment 3 (*p* = 0.006) and moment 3 to post-intervention (*p* = 0.011). In the vertical group, a significant increase was observed from pre to moment 3 (*p* = 0.020), while in the sham group, the increase from pre to post was marginal (*p* = 0.047).

HR also showed a significant effect of time (*p* = 0.020), without differences between groups (*p* = 0.226) or a significant group × time interaction (*p* = 0.192). Intra-group comparisons revealed a significant increase in HR in the side-alternating group between the pre- and moment 2 (*p* = 0.0001), followed by a further increase from moment 2 to post-intervention (*p* = 0.030). Between-group comparisons revealed significant differences only at moment 2, when the side-alternating group presented lower HR values compared to both the sham group (*p* = 0.011) and vertical group (*p* = 0.005).

### 3.5. SPPB Test Score and Hand Dynamometry

Table 4 presents the physical performance results, including scores from the SPPB test and the handgrip strength assessment.

For the SPPB balance domain, intragroup analysis before and after the intervention showed no significant differences: vertical group (4.00 ± 0.00/4.00 ± 0.00), side-alternating group (3.96 ± 0.21/3.96 ± 0.21), and sham group (4.00 ± 0.00/3.96 ± 0.20), *p* = 0.381. No significant differences were observed in the intergroup analysis (*p* = 0.327).

Regarding the gait domain, no significant changes were observed in gait speed (m/s) in the intragroup analysis: vertical group (1.03 ± 0.17/1.00 ± 0.20), side-alternating group (0.96 ± 0.15/0.98 ± 0.58), and sham group (0.97 ± 0.16/0.96 ± 0.11), *p* = 0.601; nor in the intergroup comparison (*p* = 0.266). Similarly, the 3 m walk time (s) showed no statistical differences within groups: vertical group (2.98 ± 0.48/3.15 ± 0.81), side-alternating group (3.19 ± 0.46/3.16 ± 0.58), and sham group (3.17 ± 0.50/3.17 ± 0.34), *p* = 0.394; or between groups (*p* = 0.243). The gait domain score also did not differ significantly in either the intra- or intergroup analysis: vertical group (3.96 ± 0.20/3.92 ± 0.41), side-alternating group (3.74 ± 0.45/3.70 ± 0.56), and sham group (3.79 ± 0.41/3.96 ± 0.20), *p* = 0.572; intergroup *p* = 0.064.

In the lower limb strength domain, assessed by the sit-to-stand test (time in seconds), no significant differences were found within or between groups: vertical group (11.42 ± 2.45/11.01 ± 2.88), side-alternating group (11.31 ± 3.97/11.18 ± 4.59), and sham group (11.25 ± 5.08/10.91 ± 4.62), *p* = 0.194; intergroup *p* = 0.803. Similarly, the sit-to-stand domain score showed no significant changes in either the intragroup or intergroup analysis: vertical group (3.42 ± 0.88/3.58 ± 0.78), side-alternating group (3.35 ± 1.03/3.22 ± 1.13), and sham group (3.38 ± 1.06/3.54 ± 0.88), *p* = 0.366; intergroup *p* = 0.347.

The total SPPB score also showed no significant modifications: vertical group (11.25 ± 1.07/11.54 ± 1.10), side-alternating group (11.04 ± 1.26/10.87 ± 1.42), *p* = 0.200; intergroup *p* = 0.193.

The analysis of handgrip strength in the upper limbs, also presented in Table 4, did not reveal statistically significant changes after the intervention in any of the groups. For the right arm, mean values ranged from 28.5 ± 2.69 to 28.7 ± 2.69 kgf in the sham group, from 31.6 ± 2.73 kgf to 31.2 ± 2.73 kgf in the side-alternating group, and from 32.7 ± 2.71 kgf to 32.8 ± 2.71 kgf in the vertical group. Intragroup comparisons (before vs. after the intervention) showed no significant differences (sham group: *p* = 0.7566; side-alternating group: *p* = 0.6131; vertical group: *p* = 0.8771).

Similarly, for the left arm, values ranged from 28.0 ± 2.48 kgf to 28.6 ± 2.48 kgf in the sham group, from 29.6 ± 2.52 kgf to 28.7 ± 2.52 kgf in the side-alternating group, and from 31.9 ± 2.50 kgf to 31.3 ± 2.50 kgf in the vertical group. The intragroup *p*-values were 0.4276, 0.2532, and 0.5065, respectively. The intergroup comparison after the intervention also showed no statistically significant differences for the right arm (*p* = 0.558) or the left arm (*p* = 0.679).

## 4. Discussion

### 4.1. Blood Pressure

Previous researchers demonstrated that a single session of exercise can elicit changes in physiological parameters, including reductions in BP during the recovery period [67,68]. However, the current study did not observe significant changes in SBP, DBP, or mean arterial pressure (MAP) between groups before and after the immediate WBV exercise intervention. These findings align with Aoyama et al. (2019), who reported no significant BP changes following an acute WBV exercise protocol in older adults using a frequency of 30 Hz and a peak-to-peak displacement (PPD) of 3 mm, parameters closely matching those in our study [69].

In contrast, Jinakote et al. (2023) found significant increases in SBP and MAP after acute intervention in healthy adults; however, these changes were observed across all groups evaluated (WBV exercise, WBV exercise combined with dynamic squats, and dynamic squats alone) [70]. The differences between our findings and Jinakote et al.’s results may be explained by variations in protocols. Their intervention used a lower vibration frequency (25 Hz with a PPD of 2 mm) but longer set durations (2 min per set versus 1 min in our study). Additionally, participant characteristics differ: our study involved individuals with obesity, Aoyama et al. studied older adults, and Jinakote et al. recruited younger, healthy participants, which may have influenced BP responses due to differences in health conditions.

Cardoso Jr. et al. (2010) suggest that exercise effects on clinical BP may differ between normotensive and hypertensive individuals, given altered cardiovascular dynamics in hypertension [71]. Zeigler et al. (2016) demonstrated a post-exercise hypotensive effect, a sustained BP reduction, using an acute WBV exercise protocol (mechanical vibration of 35 Hz and 5 mm amplitude) combined with resistance exercises in prehypertensive adults [72]. This combined approach produced greater BP-lowering effects compared to both a control group and a group that performed resistance exercise only [72].

Previous studies have shown that WBV exercise combined with resistance training enhanced muscle perfusion in the lower limbs and reduced peripheral vascular stiffness [73,74]. These vascular adaptations likely decrease peripheral resistance without increasing cardiac output, resulting in lower BP [75]. Discrepancies in detecting post-exercise hypotension across studies may be related to differences in the timing of BP measurements after exercise, ranging from 4 to 30 min post-exercise. Notably, Zeigler et al. (2016) reported maximum BP reductions approximately 45 min post-session [72].

### 4.2. Respiratory Rate and Arterial Oxygen Saturation

Although some studies have reported that individuals with obesity may exhibit lower tidal volume and higher RR, resulting in greater minute ventilation compared to individuals with normal body mass, participants in the present study showed a baseline RR within the normal range (12 to 20 breaths per minute) [76]. RR and peripheral SpO_2_ did not change significantly following the WBV exercise intervention. Similarly, Aoyama et al. (2019) found no changes in SpO_2_ using pulse oximetry after an acute WBV exercise protocol employing biomechanical parameters similar to those in our study (vertical VP, mechanical vibration at a 30 Hz frequency, 3 mm peak-to-peak displacement) in individuals with cardiovascular disease [69]. Supporting these findings, Sousa-Gonçalves et al. (2019) also reported no significant changes in SpO_2_ after 15 bouts of an acute WBV exercise protocol (35 Hz, 5 mm peak-to-peak displacement) in obese adolescents [77]. However, Gloeckl et al. (2017) demonstrated a significant increase in SpO_2_ following WBV exercise in individuals with chronic obstructive pulmonary disease compared to a group performing squats alone [78]. Notably, their protocol differed substantially from previous studies, including six sets of squats synchronized with auditory cues, a 10 min warm-up on a cycle ergometer, and mechanical vibration settings of 26 Hz with a 5 mm peak-to-peak displacement [78].

SpO_2_ levels during physical exertion can vary depending on the type and intensity of exercise. Monitoring SpO_2_ is essential in in-patient health management and is recommended in exercise physiology research [79]. SpO_2_ reflects the percentage of hemoglobin bound to oxygen relative to total hemoglobin [80], and hypoxemia can acutely affect critical organs such as the brain, heart, and kidneys [81]. Given the absence of WBV exercise-induced changes in SpO_2_ across cited studies, this modality may represent a low-impact, viable, and safe intervention, particularly for populations with high cardiovascular risk, such as individuals with obesity.

### 4.3. HR and Rate–Pressure Product

In the current study, HR and rate–pressure product (RPP) increased significantly after immediate WBV exercise, but only in the group performing exercises on the side-alternating VP (mechanical vibration at 30 Hz, with a peak-to-peak displacement of 2.5 mm). Conversely, Tamini et al. (2020) found no significant HR differences in obese adults after a WBV exercise protocol (30 Hz, PPD 3 mm) using a vertical VP [82]. However, Gojanovic et al. (2012), in a similar study comparing vertical VP, side-alternating VP, and control groups, corroborated our results by reporting significantly higher mean and peak HR values in the side-alternating VP group compared to the other groups [83]. Their biomechanical parameters were 26 Hz and 15 mm PPD for the side-alternating VP, and 35 Hz and 8 mm PPD for the vertical VP, tested in sedentary young women [83].

This raises the question of why side-alternating platforms elicit a greater HR response. Abercromby et al. (2007) directly compared lower limb muscle activity between side-alternating and vertical VP, finding greater overall muscle activation during side-alternating VP [84]. The asymmetric, non-vertical forces of this VP may induce increased muscle stretch, joint movement, postural challenges, and tissue vibration in the legs, contributing to distinct neuromuscular responses [85]. Therefore, it is suggested that different acute neuromuscular responses elicited by different VP types may be associated with varying cardiovascular outcomes, such as increased sympathetic activity leading to elevated HR [84].

### 4.4. Perceived Exertion (BORG Scale)

Perceived exertion can be an important factor influencing adherence to PE programs or even the performance of daily activities in overweight or obese individuals [86]. Perceived exertion changed over time in all three groups. Post hoc analysis confirms that within each group, there were significant reductions in perceived exertion, assessed by the Borg Scale (0 to 10 points), in the side-alternating, vertical, and sham groups.

Saldıran et al. (2020) [87] also reported a significant increase in Borg Scale scores in both the WBV exercise group and the control group in overweight and obese individuals. The protocol used in their study involved similar biomechanical parameters to the current study (30 Hz, low intensity); however, only a vertical VP was used [87].

Interestingly, Van Ruymbeke et al. (2014) found that frequencies that induce greater muscle activation, i.e., 20 and 30 Hz, presented lower scores on the perceived exertion scale compared to higher frequencies [88]. Furthermore, scores did not differ between breast cancer survivors and healthy controls, suggesting that the WBV exercise intervention was not perceived as more strenuous by survivors of a serious illness and may be indicated for other patients with other diseases, special health conditions with increased cardiovascular risk, or with low adherence to conventional exercise programs, such as obese participants.

### 4.5. Functionality Assessment

In the current study, functionality was assessed using the SPPB test, which evaluates balance, gait speed, and lower limb muscle strength. No statistically significant differences were observed in balance among the three groups analyzed. Similarly, Rendos et al. (2017) reported no significant improvements in balance following a single session of WBV exercise (30 Hz; PPD 5 mm) using a VP in adults with and without ankle instability [89]. Likewise, Freitas et al. (2018) found no significant changes in balance after a single session of WBV exercise (30 Hz; PPD 3 mm) on a VP in women with multiple sclerosis [90]. However, Lee et al. (2013) [91] reported significant improvements in balance following a six-week WBV exercise intervention (15–30 Hz; PPD 1–3 mm) in older adults with diabetic neuropathy. Their protocol also incorporated balance-specific exercises [91]. It is noteworthy that regular PE has the potential to effectively improve postural control by reducing body sway [92].

A potential factor contributing to the divergent findings across studies may be the duration of the intervention. In both the current study and that of Rendos et al., only a single session of WBV exercise was administered, whereas Lee et al. applied a six-week intervention that combined WBV exercise with balance intervention exercises [89,91].

The total SPPB score did not change significantly in the current study. However, Zhuang et al. (2025) [93] reported significant improvements in total SPPB scores following a 12-week WBV exercise protocol involving static squats on a VP (12 Hz; 4 mm) in older adults. It is worth noting that their study did not include a control group but rather compared outcomes with a group performing resistive exercises [93]. Similarly, Paineiras-Domingos et al. (2018) described significant improvements in SPPB scores after a protocol of 10 WBV exercise sessions using a side-alternating VP (6–14 Hz; PPD 2.5, 5.0, and 7.5 mm), when compared to a control group that performed the same exercises with the VP turned off in metabolic syndrome individuals [94]. Jo et al. (2021), in a study involving older adults, similarly demonstrated a significant increase in total SPPB scores only in the group that underwent WBV exercise intervention [27]. A vertical VP was used, with a protocol consisting of 12 intervention sessions with mechanical vibration of 10 Hz and PPD of 5 mm, followed by 20 min of resistive exercises. The control group performed only 20 min of stretching exercises [27].

It is possible that the studies by Zhuang et al. (2025), Paineiras-Domingos et al. (2018), and Jo et al. (2021) demonstrated significant improvements in SPPB scores due to the higher number of WBV exercise sessions compared to the current study, which included only a single intervention session [27,93,94].

Low physical performance is a key indicator in the diagnosis of sarcopenic obesity (SO) and has been associated with increased mortality risk. According to the Clinical Nutrition and Metabolism (ESPEN) and the European Association for the Study of Obesity, sarcopenia in SO is defined as a reduction in skeletal muscle mass and function [95]. Therefore, assessing physical performance in individuals with obesity and designing therapeutic strategies, including long-term interventions aimed at improving functional capacity, may be of great relevance for this population.

### 4.6. Handgrip Strength

In the present study, muscle strength was assessed using hand dynamometry. No significant changes were found after the intervention in any of the three groups evaluated (vertical, alternating lateral, and placebo). Corroborating our results, García-Gutiérrez (2014) also found no significant changes in hand dynamometry after a session of immediate-effect whole-body vibration (WBV) exercise in young university students who performed static squats in a vertical WBV model with biomechanical parameters of 50 Hz with a peak-to-peak displacement of 2.51 mm (High), although they used a higher frequency than that used in this study [96]. On the other hand, Santos et al. (2021) [97] reported a significant increase in intergroup values of upper limb hand dynamometry in healthy women who performed the push-up position during WBV exercise, compared to a placebo group and a group that performed static half-squats during WBV exercise. The biomechanical parameters of the WBV exercise protocol were a frequency of 45 Hz and PPD of 2 mm in a vertical VP [97]. The position adopted by the group that showed statistical significance was the “push-up” position, with hands supported on the base of the VP [97]. The difference between the results of these studies may be related to the patient’s positioning on the VP. In the present study and in that of García-Gutiérrez (2014), participants performed a static semi-squat position with their feet supported on the base of the VP [96]. In contrast, in the study by Santos et al. (2021), participants were positioned with their hands directly on the base of the VP [97].

In the study by Santos et al. (2021), one group performed a static semi-squat; in other words, this group received direct vibratory stimulation in the lower limbs, but this did not significantly affect the results of manual dynamometry after whole-body vibration (WBV) exercise, suggesting that the stimulation applied to the lower limbs was insufficient to promote efficient muscle contraction in the upper limbs or improvement in post-activation potential [97]. Physiologically, the literature offers some explanations for this improvement in performance after WBV exercise. It may be due to a phenomenon called post-activation potentiation (PAP), in which muscle performance characteristics are enhanced as a result of contractile activity preceding a physical performance test. Several mechanisms may explain PAP, such as increased recruitment of high-threshold motor units, phosphorylation of myosin regulatory light chains, and changes in the tilt angle of muscle fibers [98]. Thus, even though muscle strength and functional capacity are qualities that require chronic adaptations and longer periods of intervention like exercise, VCI exercise imposes an additional gravitational load compared to exercise without vibratory stimulation; this additional stimulus could lead to PPA. Furthermore, the body’s response to WBV exercise differs when the vibratory stimulus is applied through the feet or hands. This difference would be due to the transmission and attenuation of mechanical vibrations, which can vary depending on the body’s biomechanics and the location of the mechanical vibration source [99]. Among the study’s strengths, the randomized design stands out, with the inclusion of two distinct types of vibration platforms (vertical and side-alternating) and a sham group, which enhances the internal validity of the findings. Furthermore, the detailed assessment of physiological responses before, during, and after a single WBV exercise session allowed for a comprehensive analysis of acute changes, an aspect still little explored in the literature, especially in obese adults.

Another relevant point is the inclusion of a population with potential cardiometabolic risk and results showing no discrepant physiological changes, and the maintenance of cardiovascular parameters within limits considered safe. This reinforces the potential of WBV exercise as a viable alternative to traditional exercise programs for individuals who have difficulty adhering to them.

### 4.7. Limitations

Because this was an acute exercise protocol, the results may not reflect the chronic adaptations that occur with repeated training over time. Although it was a randomized design, the lack of a crossover format or a control group may limit the ability to fully control for interindividual variability. Furthermore, the sample was predominantly composed of female participants, which may limit the generalizability of the results to male individuals. Although this distribution partly reflects the higher prevalence of the studied condition in women, the findings should be interpreted with caution. The results were measured immediately after exercise, and it is unclear whether the observed effects persist for hours or days following the session. Future studies should investigate whether the observed acute effects translate into chronic adaptations and explore responses in more diverse populations.

## 5. Conclusions

In conclusion, considering all the findings of this study, a single whole-body vibration (WBV) exercise session promoted some immediate changes in physiological parameters and perceived exertion in obese adults, with specific responses depending on the type of vibration platform, but not on functionality. Alternating lateral WBV exercise induced significant fluctuations in heart rate and increased the heart rate–blood pressure product, although with values within the normal range. Perceived exertion increased in all groups. This suggests that, even when adding vibration to static squat exercise and increasing the gravitational load, individuals did not report significantly greater effort compared to the placebo group; furthermore, the level of exertion was not considered high. These findings may be of interest for clinical practice, since whole-body vibration exercise leads to changes in physiological parameters but does not exceed cutoff values, which may indicate that these exercises do not pose health risks. In addition, they were not considered very tiring and therefore could encourage better adherence from this population, which tends to be sedentary and has low tolerance to conventional physical exercise.

### Perspective and Facts

Obesity is a complex condition that can negatively impact the cardiovascular, respiratory, and musculoskeletal systems, increasing the risk of functional decline and cardiometabolic complications. In this context, WBV exercise emerges as a promising, low-impact option that may be particularly suitable for populations with physical limitations. The findings of this study reinforce the safety and feasibility of WBVI using alternating vertical and lateral platforms, with no adverse hemodynamic changes detected and evidence of reduced perception of exertion and preserved functionality. These results suggest that WBVI may represent a viable complementary tool for promoting physical activity in obese adults. Future studies should explore long-term adaptations and potential benefits in individuals with sarcopenic obesity or cardiometabolic comorbidities.

## Figures and Tables

**Table 1:** Baseline characteristics of study participants.

Variable	Vertical Group (*n* = 24)	Side-Alternating Group (*n* = 23)	Sham Group (*n* = 25)	*p*-Value
Age (years)	47.33 ± 8.23	41.09 ± 9.37	42.08 ± 8.95	0.055
Height (m)	1.63 ± 9.25	1.67 ± 8.68	1.63 ± 8.10	0.164
Body mass (kg)	91.67 ± 12.79	96.86 ± 13.54	93.30 ± 11.98	0.423
BMI (kg/m^2^)	34.79 ± 2.87	34.34 ± 2.94	35.04 ± 2.643	0.642
WC (cm)	108.29 ± 7.99	109.26 ± 8.19	106.31 ± 8.073	0.424
NC (cm)	39.62 ± 3.57	39.93 ± 4.19	40.08 ± 3.40	0.906
HC (cm)	117,965 ± 7.89	118.85 ± 6.36	117.14 ± 5.03	0.645
Calf C (cm)	42.20 ± 3.08	42.22 ± 3.14	42.506 ± 2.82	0.942
WHR	1.009 ± 0.070	1.026 ± 0.072	1.0197 ± 0.061	0.581
WHtR	1.512 ± 0.083	1.543 ± 0.099	1.542 ± 0.099	0.358
Fat mass (kg)	39.86 ± 7.59	39.83 ± 6.86	40.88 ± 7.01	0.864
FFM (kg)	52.45 ± 10.28	56.78 ± 12.01	52.53 ± 9.40	0.310
FM (%)	43.89 ± 5.78	41.97 ± 6.73	43.84 ± 5.89	0.480
TF (%)	42.90 ± 6.97	42.23 ± 5.77	43.83 ± 4.43	0.632
BMR (kcal)	1511.26 ± 223.48	1619.21 ± 267.27	1502.48 ± 202.19	0.166
SBP (mmHg)	125.65 ± 15.72	120.98 ± 13.79	127.91 ± 16.23	0.297
DBP (mmHg)	82.69 ± 7.69	80.50 ± 9.23	84.51 ± 11.59	0.364
Type II diabetes mellitus	1 (4%) yes 24 (96%) no	4 (17.4%) yes 19 (82.6%) no	1 (4%) yes 24 (96%) no	0.18
Male	5 (20,8%)	8 (34,8%)	7 (28%)	*p* ≥ 0.05
Female	19 (79,2%)	15 (65,2%)	18 (72%)	*p* ≥ 0.05
Systemic arterial hypertension	8 (33.3%) yes 16 (66.7%) no	4 (17.4%) yes 19 (82.6%) no	8 (32%) yes 17 (68%) no	0.43
Alcohol consumption	9 (37.5%) yes 15 (62.5%) no	14 (17.4%) yes 9 (82.6%) no	10 (%) yes 15 (%) no	0.15
Physical activity	8 (33.3%) yes 15 (66.7%) no	8 (34.8%) yes 15 (65.2%) no	6 (24%) yes 19 (76%) no	0.69
Smoking	2 (8.3%) yes 22 (91.7%) no	4 (17.4%) yes 19 (82.6%) no	3 (12%) yes 22 (88%) no	0.61
Obesity	11 (45.8%) grade1 13 (54.2%) grade 2	13 (56.5%) grade 1 10 (43.5%) grade 2	12 (48%) grade 1 13 (52%) grade 2	0.74

Abbreviation: BMI (body mass index); cm (centimeter); Calf C (calf circumference); m (meter); DBP (diastolic blood pressure); FFM (fat-free mass); FM (fat mass); HC (hip circumference); mmHg (millimeters of mercury); kg (kilogram); kcal (kilocalorie); kg/m^2^ (kilogram per square meter); BMR (basal metabolic rate); NC (neck circumference); SBP (systolic blood pressure); % (percentage); TF (trunk fat ); WBV (whole-body vibration exercise); WHtR (waist-to-height ratio); WC (waist circumference); WHR (waist-to-hip ratio). The *p*-value significance criterion was *p* ≤ 0.05.

**Table 2:** Physiological parameters in obese individuals before and after the intervention.

Variable	Vertical Group (m ± sd) *n* = 24	Intragroup *p*-Value	Side-Alternating Group (m ± sd) *n* = 23	Intragroup *p*-Value	Sham Group (m ± sd) *n* = 25	Intragroup *p*-Value	Intergroup *p*-Value	η^2^
SBP (mmHg)								
pre	125.65 ± 15.72		120.98 ± 13.79		127.92 ± 16.23			
post	126.93 ± 15.72	0.278	120.82 ± 13.25	0.919	125.17 ± 13.21	0.109	0.321	0.032
DBP (mmHg)								
pre	82.69 ± 7.69		80.50 ± 9.23		84.52 ± 11.60			
post	83.37 ± 7.15	0.405	80.31 ± 8.42	0.832	83.42 ± 11.34	0.401	0.419	0.025
MAP (mmHg)								
pre	183.08 ± 19.18		177.08 ± 19.98		187.96 ± 23.20			
post	185.01 ± 18.61	0.310	176.24 ± 18.11	0.707	183.24 ± 24.19	0.239	0.311	0.033
Pulse pressure								
pre	44.12 ± 14.95		41.43 ± 11.84		45.76 ± 12.76			
post	45.91 ± 16.90	0.411	42.47 ± 11.24	0.558	44.04 ± 12.65	0.338	0.696	0.010
RPP								
pre	9339.79 ± 1622.98		8679.47 ± 1692.74		9589.16 ± 1819.02			
post	9443.45 ± 2070.50	0.739	9395.86 ± 2273.57	0.005 *	9674.44 ± 1717.38	0.751	0.877	0.004
RR (bpm)								
pre	17.68 ± 3.00		15.53 ± 2.79		15.86 ± 4.51			
post	18.34 ± 2.89	0.154	16.50 ± 3.34	0.050	16.21 ± 4.26	0.352	0.086	0.069

Abbreviation: SBP (systolic blood pressure); mmHg (millimeters of mercury); DBP (diastolic blood pressure); MAP (mean arterial pressure); PP (pulse pressure); RPP (Rate–Pressure Product); SpO_2_ (arterial oxygen saturation); RR (respiratory rate); bpm (breaths per minute); *n* (total number of individuals); % (percentage); WBV exercise (whole-body vibration); VP (vibrating platform). Statistics presented as mean ± standard deviation; intragroup, intergroup *p*-values; Eta squared (η^2^); *p* value significance criterion was * *p* ≤ 0.05.

**Table 3:** Physiological parameters (HR, SpO_2_) and perception of effort (Borg scale) during the intervention.

Variable	Group	Pre	Moment 2 (5th Set)	Moment 3 (10th Set)	Post	Intragroup *p*-Value	Intergroup *p*-Value
SpO_2_	Sham	98.2 ± 1.2	98.2 ± 0.9	98.2 ± 1.2	98.1 ± 1.0	0.149	0.229
	Vertical	96.1 ± 1.1	98.4 ± 0.8	98.5 ± 0.7	98.4 ± 0.7		
	Side-alternating	97.7 ± 1.0	98 ± 1.2	98 ± 1.3	97.9 ± 1.2		
Borg scale	Sham	1.8 ± 2.3	2.12 ± 1.5	2.5 ± 1.8	2.7 ± 1.8	<0.05	0.804
	Vertical	1.5 ± 2.0 *	1.9 ± 1.9 *	2.6 ± 2.3 *	2.6 ± 2.5		
	Side-alternating	1.4 ± 2.2 *	1.9 ± 2.0 *	2.3 ± 2.2 *	2.1 ± 2.3 ^#^		
HR	Sham	73.6 ± 11.33	89.0 ± 13.6	91.2 ± 14.6	94 ± 13.8	<0.01	0.226
	Vertical	70.3 ± 8.8	94.8 ± 14.3	94.8 ± 16.2	91.1 ± 19		
	Side-alternating	73.7 ± 12.5	85.4 ± 13.4 **^¥^	91.2 ± 18.3	83.7 ± 16.8 ^§^		

SpO_2_, arterial hemoglobin oxygen saturation; HR, heart rate. * *p* < 0.05 pre to moment 3; ^#^ *p* < 0.05 moment 3 to post; ** *p* < 0.01 pre to moment 2; ^¥^ *p* < 0.01 moment 2 side-alternating vs. moment 2 sham and vertical; ^§^ pre- to post-intervention 2.

**Table 4:** Short Physical Battery test and handgrip strength.

Variable	Vertical Group (m ± sd) *n* = 24	Side-Alternating Group (m ± sd) *n* = 23	Sham Group (m ± sd) *n* = 25	Intragroup *p*-Value	Intergroup *p*-Value
Balance (score)					
pre	4.00 ± 0.00	3.96 ± 0.21	4.00 ± 0.00	0.381	0.327
post	4.00 ± 0.00	3.96 ± 0.21	3.96 ± 0.20		
Gait speed (m/s)					
pre	1.03 ± 0.17	0.96 ± 0.15	0.97 ± 0.16	0.601	0.266
post	1.00 ± 0.20	0.98 ± 0.20	0.96 ± 0.11		
3 m Gait (s)					
pre	2.98 ± 0.48	3.19 ± 0.46	3.17 ± 0.50	0.394	0.243
post	3.15 ± 0.81	3.16 ± 0.58	3.17 ± 0.34		
3 m Gait (score)					
pre	3.96 ± 0.20	3.74 ± 0.45	3.79 ± 0.41	0.572	0.064
post	3.92 ± 0.41	3.70 ± 0.56	3.96 ± 0.20		
Stand to sit (s)					
pre	11.42 ± 2.45	11.31 ± 3.97	11.25 ± 5.08	0.194	0.803
post	11.01 ± 2.88	11.18 ± 4.59	10.91 ± 4.62		
Stand to sit (score)					
pre	3.42 ± 0.88	3.35 ± 1.03	3.38 ± 1.06	0.366	0.193
post	3.58 ± 0.78	3.22 ± 1.13	3.54 ± 0.88		
SPPB Total (score)					
pre	11.25 ± 1.07	11.04 ± 1.26	11.17 ± 1.24	0.200	0.193
post	11.54 ± 1.10	10.87 ± 1.42	11.46 ± 0.98		
Handgrip strength (kgf)					
Right hand				0.877	
pre	32.7 ± 2.71	31.6 ± 2.73	28.5 ± 2.69	0.613	0.558
post	32.8 ± 2.71	31.2 ± 2.73	28.7 ± 2.69	0.756	
Left hand				0.506	
pre	31.9 ± 2.50	29.6 ± 2.52	28.0 ± 2.48	0.253	0.679
post	31.3 ± 2.50	28.7 ± 2.52	28.6 ± 2.48	0.427	

Abbreviation: VP (vibrating platform); m/s (meters per second); s (seconds); SPPB (Short Physical Battery test), total score range from 0 to 12. Statistics presented as (m ± sd) mean ± standard deviation; intragroup, intergroup *p*-values; *p* value significance criterion was *p* ≤ 0.05.

## Data Availability

The data presented in this study are available on reasonable request from the corresponding author. The data are not publicly available due to ethical and privacy restrictions.
